# Complete Genome Sequence of Bacteriophage vB_Hercules_Set, Which Infects Enteric Pathogen Salmonella enterica Serovar Typhimurium

**DOI:** 10.1128/mra.01282-22

**Published:** 2023-03-16

**Authors:** Evan N. Bennett, Ibrahim Ayyash, Joshua Hughes, Teagen Comeau, Shallee T. Page

**Affiliations:** a College of Health and Natural Sciences, Franklin Pierce University, Rindge, New Hampshire, USA; Queens College Department of Biology

## Abstract

We report the isolation, sequencing, and annotation of bacteriophage vB_Hercules_Set, a kuttervirus infecting human pathogen Salmonella enterica. vB_Hercules_Set was isolated from a slurry of soil and deli meat collected in New Hampshire in 2021. The genome length is 157,338 nucleotides, containing 210 protein-coding genes and five tRNAs.

## ANNOUNCEMENT

Salmonella enterica serovar Typhimurium is a pathogen in humans and other animals, a common culprit of gastrointestinal disease from contaminated food (1). Over 40,000 cases of food poisoning are reported in the United States each year, and globally, nontyphoidal Salmonella is estimated to kill over 150,000 people a year (2).

Here, we describe the isolation of a double-stranded DNA bacteriophage, vB_Hercules_Set, which infects Salmonella enterica serovar Typhimurium (3, 4). vB_Hercules_Set was extracted from a slurry of moist soil and deli meat in September 2020 at Rindge, NH, USA. The extract was washed with nutrient broth, and bacteriophages were extracted through a 0.22-μm filter. The filtrate was enriched by coculture with Salmonella enterica serovar Typhimurium (Carolina Biological) and then overlaid with S. enterica serovar Typhimurium in 0.4% nutrient broth agar. Plaques were ~1 mm in size and mildly turbid after 24 h at 37°C. Purification was accomplished by two rounds of 10-fold dilutions and plating from a single plaque. Amplification was achieved by soaking three overlay plates in buffer (10 mM Tris, pH 7.5, supplemented with 10 mM MgSO_4_, 68 mM NaCl, and 1 mM CaCl_2_) (4).

Double-stranded genomic DNA was isolated from vB_Hercules_Set using the Quick-DNA viral kit (Zymo) and quantitated by a NanoDrop spectrophotometer (ThermoFisher) and assessed by a Qubit fluorometer (ThermoFisher). Library preparation, sequencing, and assembly were performed at the North Carolina State University Genomic Sciences Laboratory (NCSUGSL). The DNA was assessed using ScreenTape (Agilent), prepared for sequencing using the TruSeq DNA Nano library prep kit (Illumina) via random fragmentation, and subjected to bead-based size selection followed by ligation of dual-index adapters in multiplex runs. Pair-end sequencing (Illumina NovaSeq6000) yielded 3.38 × 10^6^ reads of an average length of 150 nucleotides (nt) and ~3,240-fold coverage of the genome.

Software was run using default settings except where otherwise noted. Assessment of read quality, adapter trimming, and assembly were done using the CLC Genomics Workbench v.21 assembly tool v.6.5.1 (Qiagen) at NCSUGSL.

Autoannotation was performed at Franklin Pierce University with DNA Master v.5.23.6 (5), GLIMMER v.3.02 (6), and GeneMark v.3.25 (7). Annotation was then compared to GeneMarkS v.4.28 using prokaryotic and virus models. Predicted models for start sites were then carefully refined manually using predicted coding potential, ribosome binding site scores, and alignment to related phages from the abovementioned software and BLASTp. Putative functions were identified based on BLASTp alignment (nr database), synteny with related bacteriophages, and HHPred (8). tRNA predictions from Aragorn v.1.2.38 (9) and from tRNAscan-SE v.2.0 (10) were trimmed manually (11).

**FIG 1:**
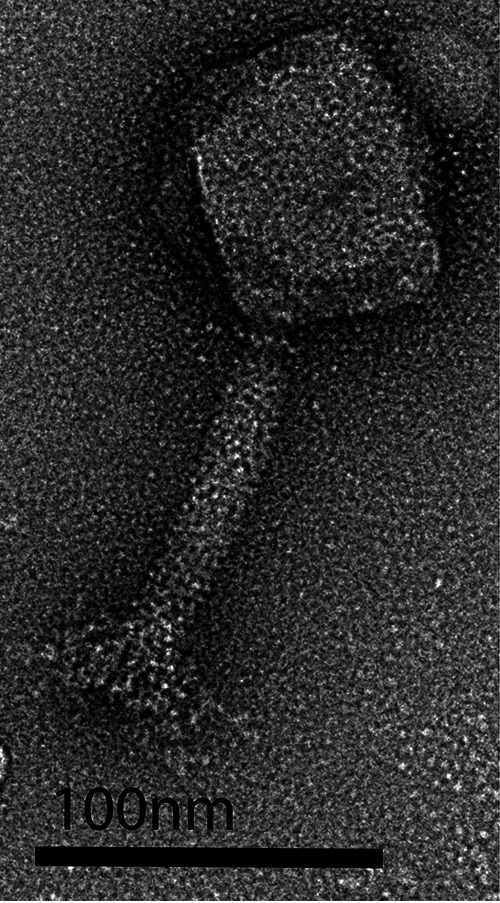
Transmission electron micrograph of vB_Hercules_Set. Bar, 100 nm. The capsid was estimated at 66 nm, and the tail was estimated at 117 nm.

vB_Hercules_Set was identified as a kuttervirus based on nucleotide similarity. Its closest relatives are Salmonella phage Vi01 and Escherichia phage vB_EcoM_Sa157lw (each with >98% identity and >92% coverage). Negative staining with 1% uranyl acetate (12) was visualized via transmission electron microscopy (TEM) at Dartmouth University. TEM revealed a tail length of ~117 nm and a capsid of 66 nm (Fig. 1). A summary can be found in Table 1. Notable predicted genes include a pro-head protease and core protein and DNA primase sequences separated by six open reading frames.

**TABLE 1:** Characteristics of vB_Hercules_Set

Characteristic	Value for vB_Hercules_Set
GenBank accession no.	OP423032
Genome size	157,338 nt
Collection location (coordinates)	42.77955699 N, 72.05625289 W
Sequencing coverage (fold)	3,240
GC content (%)	44.9
No. of protein-coding genes	205
No. of tRNAs	5
No. of tmRNAs	0
Plaque size (mm)	1
Capsid size (nm)	66
Tail length (nm)	117

### Data availability.

vB_Hercules_Set is available at GenBank under accession no. OP423032.1, BioProject no. PRJNA889411, BioSample no. SAMN31243182, and Sequence Read Archive no. SRR21863082.
